# Mitochondrial respiration in highly aerobic canines in the non-raced state and after a 1600-km sled dog race

**DOI:** 10.1371/journal.pone.0174874

**Published:** 2017-04-26

**Authors:** Benjamin Miller, Karyn Hamilton, Robert Boushel, Katherine Williamson, Verena Laner, Erich Gnaiger, Michael Davis

**Affiliations:** 1 Department of Health and Exercise Science, Colorado State University, Fort Collins, CO, United States of America; 2 School of Kinesiology, University of British Columbia, Vancouver, British Columbia, Canada; 3 Waypoint Veterinary Education, Edmond, Oklahoma, United States of America; 4 Oroboros Instruments, Innsbruck, Austria; 5 Department of Visceral, Transplant and Thoracic Surgery, D. Swarvoski Research Laboratory, Medical University of Innsbruck, Innsbruck, Austria; 6 Department of Physiological Sciences, Oklahoma State University, Stillwater, Oklahoma, United States of America; University of Birmingham, UNITED KINGDOM

## Abstract

At the annual Iditarod Race, Alaskan Huskies repeatedly run for up to 8 hours at 16 km/h to complete 1600 km. We previously demonstrated high rates of mitochondrial protein synthesis in Alaskan Huskies, which we suspected allowed rapid remodeling of mitochondrial proteins in response to energetic stress. The purpose of this study was to examine mitochondrial respiration in permeabilized skeletal muscle fibers of Alaskan Huskies in the offseason (Non-raced) and following the 1600 km Iditarod Sled Dog Race (Raced). We hypothesized that compared to Non-raced Huskies, raced Huskies that completed a 1600 km race would have greater mitochondrial respiratory capacities, and improvements in capacities of oxidative phosphorylation (OXPHOS) based on NADH-generating substrates as compared to fatty acids. Using high-resolution respirometry (HRR) we investigated the respiration of permeabilized muscle fibers from Alaskan Huskies. Maximum capacities were 254±26 pmol^.^s^-1.^mg^-1^ for OXPHOS (coupled, P) and 254±37 pmol^.^s^-1.^mg^-1^ for the electron transfer system (ETS; non-coupled, E). After racing respiratory capacities from NADH-linked substrates, but not fat-derived substrates increased. Finally, the OXPHOS to ETS capacity ratio (P/E) increased after racing from 0.90±0.03 to 0.97±0.02. From our previous studies and the current study, we conclude that Alaskan Huskies maintain high mitochondrial protein turnover to facilitate rapid adaptation to environmental extremes and energetic challenges.

## Introduction

At the annual Iditarod Race, winning sled dog teams can cover 1600 km in less than nine days. Impressively, the dogs repeatedly complete runs of up to 8 hours at 16 km/h. Although difficult to measure, unpublished data indicate that endurance trained Alaskan Huskies have V_O2max_ values around 240 ml∙min^-1^∙kg^-1^, which are significantly above the body-mass specific V_O2max_ prediction for mammalian species [1,2]. It therefore follows that Alaskan Huskies might have peripheral mitochondrial adaptations that allow for high work rates over prolonged periods of time.

The mitochondrial surface area in the skeletal muscle of Labrador Retrievers has been shown to be twice as large as a similarly sized goat, which is a less aerobic species [3]. Although a well-developed mitochondrial reticulum is a requisite for high aerobic capacity, mitochondrial content does not necessarily equate with mitochondrial remodeling [4]. Importantly, it is possible to have a high rate of mitochondrial protein turnover, indicative of mitochondrial remodeling, with no change in mitochondrial content [4]. Therefore, it is important to assess outcomes beyond mitochondrial content when considering exercise adaptations.

We recently assessed mitochondrial protein turnover in a group of Alaskan Huskies in a relatively untrained state and at the onset of an exercise-training program [5]. Given the high exercise capacity of Alaskan Huskies, we hypothesized that at the onset of the exercise-training program, the dogs would have a large increase in mitochondrial biogenesis. We were surprised to find that there was no difference in mitochondrial protein synthesis between dogs not engaged in exercise training (sedentary) and dogs initiating an exercise training program. However, the sedentary dogs had four times the resting rate of mitochondrial protein synthesis of humans and double that of humans who are undergoing exercise training [5]. These data illustrate the extremely high mitochondrial protein synthetic rates, even when not training, in Alaskan Huskies. We speculate that this high turnover rate is excess mitochondrial biogenic capacity that could facilitate changes in mitochondrial function to exercise despite no further increases in protein synthesis, helping to explain the rapid and comprehensive adjustment to extreme environmental changes previously reported in dogs, which dogs do both quickly and comprehensively [6–8].

In a recent study we found that, contrary to what is commonly assumed, exercising Alaskan Huskies were highly reliant on carbohydrate for energy production during exercise [8]. In fact carbohydrate use exceeded fatty acid oxidation (FAO) during a bout of mild-to-moderate intensity exercise, a finding that is documented in previous literature on Labrador Retrievers [9], but often overlooked. From our study, it appears that fat stores make glycerol available to sustain gluconeogenesis and carbohydrate oxidation [8]. By this mode of energy provision, fat stores are still used, although they are used to sustain carbohydrate oxidation and potentially maintain heat production by futile cycling mechanisms. Although initially counterintuitive, it is possible that maintaining energy production in this manner allows for higher sustained work rates, since NADH/FADH2 is higher during carbohydrate oxidation than fat oxidation resulting in improved ATP synthesis to oxygen consumption [10]. It is possible, although not tested, that fatty acids are then primarily oxidized during resting periods [11]. If true, one would predict mitochondrial adaptations to promote greater carbohydrate substrate use during exercise.

In addition to mitochondrial biogenesis–and particularly in the absence of mitochondrial biogenesis—changes in cellular energy production can be evaluated by assessing mitochondrial function. Mitochondrial function represents the integrated outcome of mitochondrial remodeling and the local mitochondrial environment. An increasingly utilized assessment of skeletal muscle mitochondrial function is high-resolution respirometry in permeabilized muscle fibers. This approach assesses mitochondria in their native conformation, although it does not allow selective separation of mitochondria for which isolation protocols would be required [12]. The capacity to assess mitochondrial function in permeabilized fibers has been greatly enhanced by the development of high-resolution respirometry [12], which has been used in a variety of species [13].

There are fundamental species differences in mitochondrial bioenergetics [14]. Understanding these differences could provide insight into the important adaptations that allow different species, or strains, to succeed when faced with environmental stresses. There has been heavy selection pressure to account for the diversity apparent in the 300+ recognized dog breeds, especially given that the domestication of canis lupus familiaris only happened approximately 27,000 years ago [15]. Therefore, the Alaskan Husky, a breed that has been selected for sustained exercise, might exemplify the adaptive potential of mitochondrial function in mammalian species.

The purpose of this study was to examine mitochondrial respiration in permeabilized muscle fibers of Alaskan Huskies in the offseason (Non-raced) and following the 1600 km Iditarod Sled Dog Race (Raced). Further, due to our previous findings about substrate preferences during exercise [8], we examined substrate-specific changes in respiratory capacity. We hypothesized that compared to Non-raced Huskies, Raced Huskies that completed a 1600 km race would have improvements in oxidative phosphorylation (OXPHOS) capacities, and the improvements in maximal respiratory capacities would be primarily via increase capacity to oxidize carbohydrate compared to fat-derived substrates.

## Materials and methods

Overall study design: All procedures were approved by the Oklahoma State University IACUC prior to the start of the experiments, and informed consent was obtained from the owner of the dogs prior to the study. Alaskan Huskies were tested at three different times at a competitive racing kennel in Alaska. The kennel is home to teams that boast multiple Iditarod wins and over 30 years of elite endurance competitions. The first study took place in January 2014 and was used to establish dog-specific experimental protocols. Given the preliminary nature of these studies, the data are not reported here but findings from that study informed the current design. The second study took place in March 2014 immediately following the Iditarod sled dog race with the data reported here as Raced. The final study took place in August 2014, the offseason from racing, when the dogs had not been undergoing regular exercise training for the previous five months. The dogs studied in August are, therefore, referred to as Non-raced.

Dog characteristics: The Non-raced group contained five dogs; 3 males and 2 females. The dogs were 3–5 yrs of age (mean±SD = 4.0±0.7 yrs) and weighed 27±4 kg. The Raced group contained six dogs, which were all male. The dogs were 2–5 yrs of age (mean±SD = 3.8±0.6 yrs) and weighed 24±3 kg. Three dogs were in both groups. All dogs (both Raced and Non-raced) underwent approximately 7 months of exercise conditioning immediately prior to the March 2014 Iditarod, and all had been rested with no compulsory exercise for the 4.5 months prior to samples being collected in August 2014. All dogs were fed a commercial dog food formulated for athletic activity (30% crude protein, 20% crude fat on an as-fed basis) with volume adjusted for each dog to maintain normal body weight and a Body Condition Score of 4/9. During intensive exercise sessions such as prolonged training runs or when racing, animal-derived foodstuffs (raw fish, poultry skins, tripe) were added to the meals to increase palatability, but accounted for less than 10% of the total caloric intake. Conditioning consisted of group exercise in harness, with speed and duration of the individual exercise sessions progressively increased as allowed by environmental factors and subjective fitness of the dogs. Such training typically involves 3 to 4 days of running per week, starting at 15–20 km per day and increasing as conditioning and weather allows. Cumulative training distance for the average dog is approximately 4500 km, with individual training runs of over 125 km during the month prior to the Iditarod Sled Dog Race.

Biopsy procedure: Skeletal muscle samples were collected from all dogs following an overnight fast. Raced dogs were sampled 72–120 h after finishing the Iditarod Sled Dog Race. Non-raced dogs did not undergo any exercise training for 5 months prior to sampling. Anesthesia was induced with an intravenous bolus of propofol (5 mg/kg). Anesthetic depth was monitored by heart rate, respiratory rate, pulse oximetry and response to pain. Adequate anesthetic depth was maintained with additional intravenous boluses of propofol. The use of a general anesthesia avoided potential problems of local anesthetics [13]. Hair was clipped from a 2 cm x 2 cm site over the middle of the biceps femoris muscle with the guard hairs taped back. The biopsy site was aseptically prepared and draped. An incision was made in the skin with a #15 scalpel blade and a commercial biopsy needle (12 gauge E-Z Core single action biopsy needle, Products Group International, Lyons CO) was inserted through the stab incision into the belly of the muscle. With a maximum of 3 passes of the needle, roughly 20–30 mg of tissue wet weight, W_w_, was obtained. Biopsies were immediately transferred into vials with ice-cold BIOPS solution (2.77 mM CaK_2_-EGTA, 7.23 mM K_2_-EGTA, 20 mM imidazole, 20 mM taurine, 50 mM K-MES, 0.5 mM dithiothreitol, 6.56 mM MgCl_2_, 5.77 mM ATP, and 15 mM phosphocreatine, adjusted to pH 7.1) [12]. The vials containing the muscle were kept on ice until further processing. Dogs recovered from anesthesia on padded bedding under the direct supervision of a veterinarian.

Preparation of permeabilized fibers: Permeabilization was performed according to previously published methods [12]. Under a magnifying light, muscle fibers were mechanically dissected to achieve a high degree of fiber separation. We anticipated a high mitochondrial respiratory capacity and therefore the fiber bundle volume dissected and separated per sample was small (~1 mg W_w_). The muscle fibers were highly pliable and supple, containing an intricate, very elastic network of connective tissue between fibers. Once pallor of the muscle samples was achieved in the dissection process, careful distinction, separation and removal of connective tissue was achieved using a stereomicroscope. The muscle bundles were then subjected to 30 min of gentle agitation at 4°C in 2–4 ml of BIOPS solution containing 50 μg/ml saponin. The permeabilized fiber bundles were then rinsed for 10 min with gentle agitation in ice-cold mitochondrial respiration medium (MiR06Cr; 0.5 mM EGTA, 3 mM MgCl_2_, 60 mM K-lactobionate, 20 mM taurine, 10 mM KH_2_PO_4_, 20 mM HEPES, 110 mM sucrose, 1 g/l BSA essentially fatty acid free, 280 u/ml catalase, and 20 mM creatine, adjusted to pH 7.1). Permeabilized muscle fibers were then blotted dry on a filter paper, weighed (Microbalance, XS105DU, Mettler Toledo Intl. Inc., Columbus, OH), and 0.8–1.2 mg W_w_ were immediately added to each chamber of six Oxygraph-2k instruments (OROBOROS INSTRUMENTS, Innsbruck, Austria) for respirometry measurements.

High-resolution respirometry: High-resolution respirometry measurements were performed in a two-chamber system with six instruments (12 chambers) in parallel. The instruments were calibrated daily for background oxygen consumption, back-diffusion of oxygen into the chamber, oxygen leak to the exterior, and sensor oxygen consumption [12]. Triplicate samples from two dogs were investigated on each experimental day. Measurements with permeabilized fibers were made within an oxygen range of 500 to 250 μmol^.^ml^-1^ to avoid O_2_ diffusion limitation, and were performed at 37°C. The use of catalase in the respiration media was necessary in order to maintain high pO_2_ in the reaction chamber, but precluded the determination of the effects of reactive oxygen species in this highly aerobic tissue.

**Fig 1:**
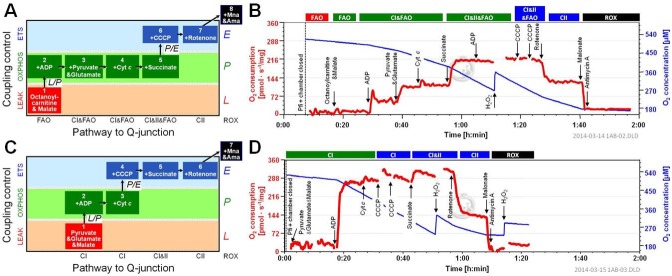
Conceptual schematics of substrate-uncoupler-inhibitor titration (SUIT) protocols for ex vivo respirometry in permeabilized muscle fibers. SUIT1 (Fig 1A) was designed to primarily examine control of OXPHOS capacity. SUIT2 (Fig 1C) was designed to measure respiration with NADH-linked substrates and succinate (S) without fatty acids. Non-phosphorylating respiration in the absence of adenylates is indicated by leak respiration (LEAK or L). Coupled respiration with ADP is represented by oxidative phosphorylation (OXPHOS or P). Uncoupling titrations with CCCP yield assessment of electron transfer system capacity (ETS or E). Substrate additions of octanoylcarnitine (Oct) and malate (M) are indicative of fatty acid oxidation (FAO). Additions of substrates pyruvate (P) and glutamate (G) are indicative of respiration with additive electron supply by NADH to CI in the presence of fatty acid substrates (SUIT1) or the absence of fatty acid substrates (SUIT2). Subsequent addition of S is indicative of convergent electron supply to the Q-junction through CII. Succinate-linked respiration is indicated by inhibition of CI by rotenone (Rot). Finally, residual oxygen consumption (ROX) is determined after inhibition of CIII with malonate (Mna) and antimycin A (Ama). Representative edited examples of each protocol in Raced dogs are shown in Fig 1B and 1D, respectively.

Two different Substrate-Uncoupler-Inhibitor Titration (SUIT) protocols were applied for selection and combination of substrate types to fuel the electron transfer system (ETS) on the pathway level of 1) NADH- and FADH_2_-linked dehydrogenases including the TCA cycle and beta-oxidation, and 2) electron transfer complexes converging at the Q-junction and downstream complexes (C) III&IV. SUIT1 focused on substrate control after stimulation of fatty acid-linked respiration by ADP with sequentially added NADH-linked substrates and succinate (CII), followed by uncoupler titrations. SUIT2 was designed to investigate mitochondrial respiration without fat substrate supply and focused on coupling control with primarily NADH-linked substrate sources (Fig 1).

The substrates pyruvate (P), glutamate (G) and malate (M) generate NADH through their respective dehydrogenases in the TCA cycle and provide electrons to CI of the ETS with subsequent transfer to the Q-junction and CIII&IV. Succinate (S), through its dehydrogenase located on the inner side of the mitochondrial membrane, feeds electrons directly upstream of the Q-junction via FADH_2_. Octanoylcarnitine (Oct) is a medium-chain fatty acid that generates membrane-bound FADH_2_ in β-oxidation providing electrons to the Q-junction through electron transferring flavoprotein, and also generates NADH supplying CI and transfer to the Q-junction. Malate (M) is also a necessary co-substrate for oxidizing fatty acids through its oxidation to oxaloacetate, which further stimulates acetyl CoA entry into the TCA cycle and, if accumulated, inhibits fatty acid oxidation. Carbonyl cyanide m-chloro phenyl hydrazone (CCCP) is a protonophore that uncouples respiration from ATP synthesis by collapsing the gradient, with maximum flux at optimum CCCP concentration, indicated as the non-coupled state E. Rotenone (Rot) is a CI inhibitor, which also completely inhibits fatty acid oxidation (FAO) allowing for isolation of CII and downstream complex respiration. Malonate (Mna) blocks electron transfer through CII to inhibit S driven respiration. Antimycin A (Ama) inhibits CIII and thus prevents ETS linked respiration.

SUIT1 included: M (0.5 mM), Oct (0.2 mM), ADP (6.2 mM), P (5 mM), G (10 mM), cytochrome c (c, 10 μM), S (10 mM), CCCP (in one or multiple 0.5 μM steps), Rot (0.5 μM), and Ama (2.5 μM) and Mna (5 mM). SUIT2A included: M (0.5 mM), P (5 mM), G (10 mM), ADP (6.2 mM), c (10 μM), CCCP (in 0.5 μM steps), S (10 mM), Rot (0.5 μM), and Ama (2.5 μM) and Mna (5 mM). In the non-raced dogs, the order of S and CCCP titrations were reversed (SUIT2B).

Analysis: Each muscle biopsy sample was divided into three portions of ~1 mg and triplicate measurements of oxygen flux were measured for each SUIT protocol. Oxygen flux in the individual chambers was calculated as the slope of the oxygen concentration measurement (multiplied by -1 to result in a positive value) with a data-sampling interval of 2 seconds. The mean oxygen flux for each step of the respective SUIT protocol was then calculated using a timespan of at least 30 seconds immediately before the next SUIT protocol titration. The replicate independent values of each functional outcome (e.g. CI, CII, etc.) of an individual dog were averaged to provide a single value for that dog for a given SUIT protocol. However, in some cases an individual run was rejected during the experiment if the oxygen flux increased more than ~30% after addition of cytochrome c. The validity of this cutoff point was determined from a previous analysis of canine muscle samples that demonstrated no relationship between CI-linked OXPHOS nor CI&II-linked ETS capacity and the flux control factor (FCF_c_, the increase of OXPHOS capacity after addition of 10 μM c normalized for c-stimulated respiration) [16]. In the muscle samples in which FCFc was under 0.30, any apparent damage caused by the mitochondria preparation could be rescued by addition of 10 μM c, thus restoring flux capacities comparable to samples with no damage. The effects of training status were evaluated using a Student’s T-test, with p<0.05 considered statistically significant. Values are presented as means ± SD.

## Results

**Fig 2:**
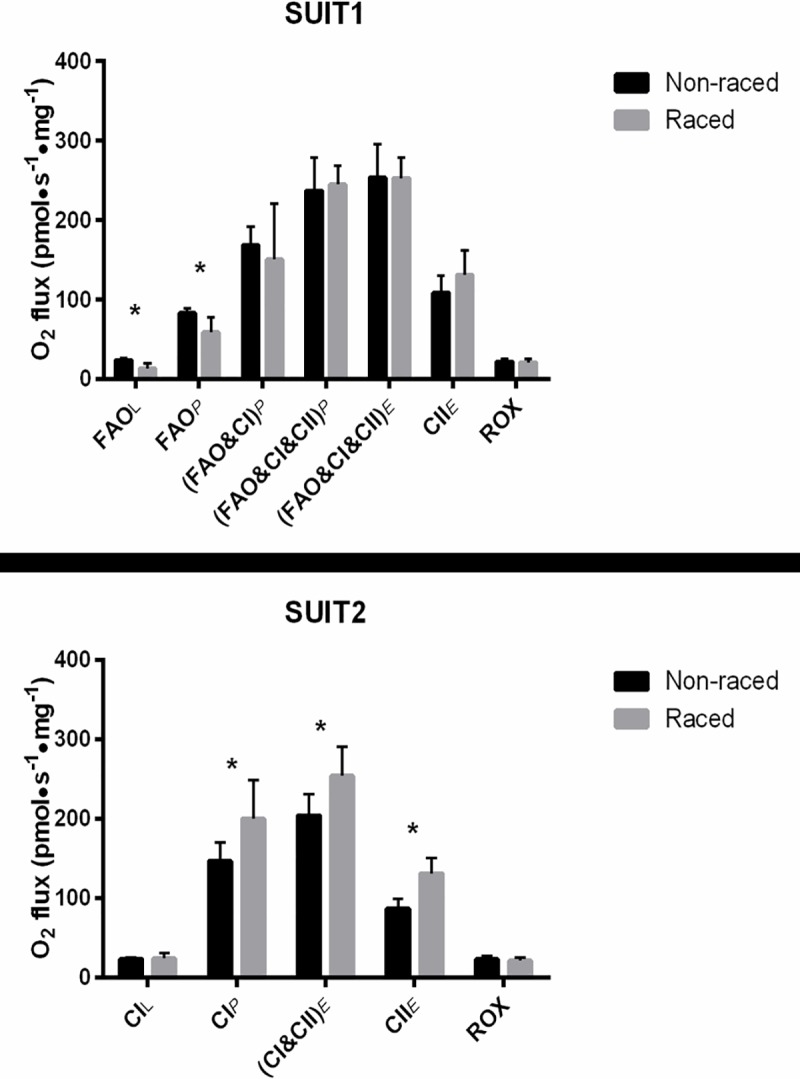
Mitochondrial respiration presented as oxygen flux (O_2_ flux, pmol^.^s^-1.^mg^-1^) in skeletal muscle in Non-raced and Raced Alaskan Huskies. Values for SUIT1 (A) are indicative of leak respiration (FAO_L_), oxidative phosphorylation with fatty acid derived substrates at CI (FAO_P_), and CI and CII ((CI&CII&FAO)_P_); electron transfer system (ETS) capacity with CI and CII substrates ((CI&CII&FAO)_E_), CII only (CII_E_), or CI only ((CI&FAO)_E_); and residual oxygen consumption (ROX). Values for SUIT2 (B) were assessed in the absence of FAO substrates and were indicative of NADH-linked substrates only. In the Raced dogs there was a significant decrease in both FAO_L_ and FAO_P_ (A). However, when only NADH-linked substrates were provided (B), Complex (C) I phosphorylation (CI_P_), uncoupled CI and CII ((CI&CII)_E_), and CII only (CII_E_) respiration are significantly greater in the Raced compared to Non-raced dogs. N = 5 (Non-raced) and 6 (Raced). *p<0.05.

Fig 2A and 2B show the mitochondrial oxygen flux responses to sequential addition of substrates for the SUIT1 and SUIT2 protocols respectively. All values are group mean ± SD values for the dogs in the offseason following 5 months without training (Non-raced) compared to immediately (within 1 week) after the 1600 km Iditarod Race (Raced).

**Fig 3:**
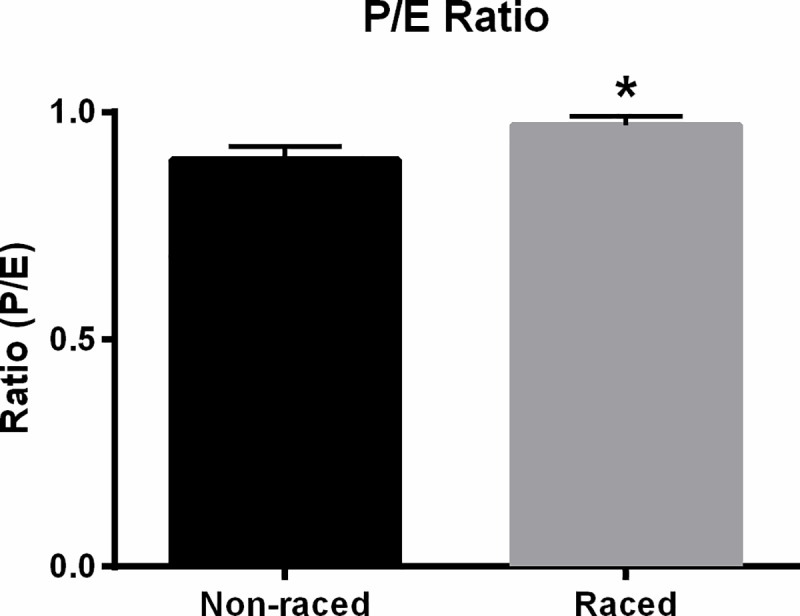
Ratio of oxidative phosphorylation capacity (P) to electron transfer system capacity (E) in Non-raced and Raced Alaskan Huskies when FAO and NADH-linked substrates are provided (SUIT1). The P/E ratio in the Raced is significantly greater than Non-raced dogs in SUIT1, indicating a closer matching of the capacity of the phosphorylation system with ETS capacity. N = 5 (Non-raced) and 6 (Raced). *p<0.05.

SUIT1: Mitochondrial oxygen flux with fatty acid substrate supply are shown in Fig 2A. LEAK respiration (FAO_L_) representing non-phosphorylating respiration in the absence of adenylates, measured with the addition of M and Oct was lower in Raced (14±7) compared to Non-raced (24±2 pmol^.^s^-1.^mg^-1^) dogs (p < 0.005). Addition of ADP to achieve OXPHOS capacity with fatty acid substrate (FAO_P_) resulted in FAO_P_ that was lower in Raced (59±19) compared to Non-raced (83±6 pmol^.^s^-1.^mg^-1^) dogs (p = 0.01). There were no differences in coupled respiration with the addition of the NADH-linked substrates G and P ((CI&FAO)_P_) between Raced (151±70 pmol^.^s^-1.^mg ^-1^) and Non-raced (168±24) dogs (p = 0.30). There was also no difference in oxidative phosphorylation capacity with convergent electron supply to the Q-junction after addition of S (CI&CII&FAO)_P_ between Raced (245±24) and Non-raced (237±42 pmol^.^s^-1.^mg ^-1^) (p = 0.34). The parity of additive respiratory flux was confirmed with substrate control ratios (CI/CI&II) for Non-raced (0.71±0.05) and Raced (0.63±0.24) conditions (data not shown). Uncoupling of the respiratory system with subsequent titration of CCCP (CI&CII&FAO)_E_ increased oxygen flux above coupled respiration in both the Raced (253±26) and Non-raced (254±42, pmol^.^s^-1.^mg^-1^) dogs (p = 0.49), as reflected in the P/E ratio that was higher in Raced (0.97±0.02) compared to Non-raced (0.90±0.03) (p < 0.005) (Fig 3). When isolating the respiratory flux with electron flow through CII and downstream with addition of Rot to block CI (CII_E_), no differences were observed between Raced (132±31) and Non-raced (109±22 pmol^.^s^-1.^mg^-1^) (p = 0.10).

SUIT2: Mitochondrial oxygen flux without fatty acid substrate supply is shown in Fig 2B. ADP-stimulated respiration with NADH-generating substrates to CI (CI_P_) was higher in Raced (200±49) compared to Non-raced (147±23, pmol^.^s^-1.^mg^-1^) (p = 0.03). The maximal ETS capacity with NADH and the addition of S generated FADH_2_ for convergent electron supply to the Q-junction through CI&II ((CI&CII)_E_) was also higher in Raced (254±37) compared to Non-raced (204±27 pmol^.^s^-1.^mg^-1^) dogs (p = 0.02). The respiratory flux with S after inhibition of CI by Rot (CII_E_) was higher in Raced (132±19) compared to Non-raced (87±11 pmol^.^s^-1.^mg^-1^) (p < 0.005), however the oxygen flux for CI from the loss of O_2_ flux by the addition of Rot (CI_E_) was similar between groups (Raced, 123±31; Non-raced, 117±17 pmol^.^s^-1.^mg^-1^) (p = 0.37).

**Fig 4:**
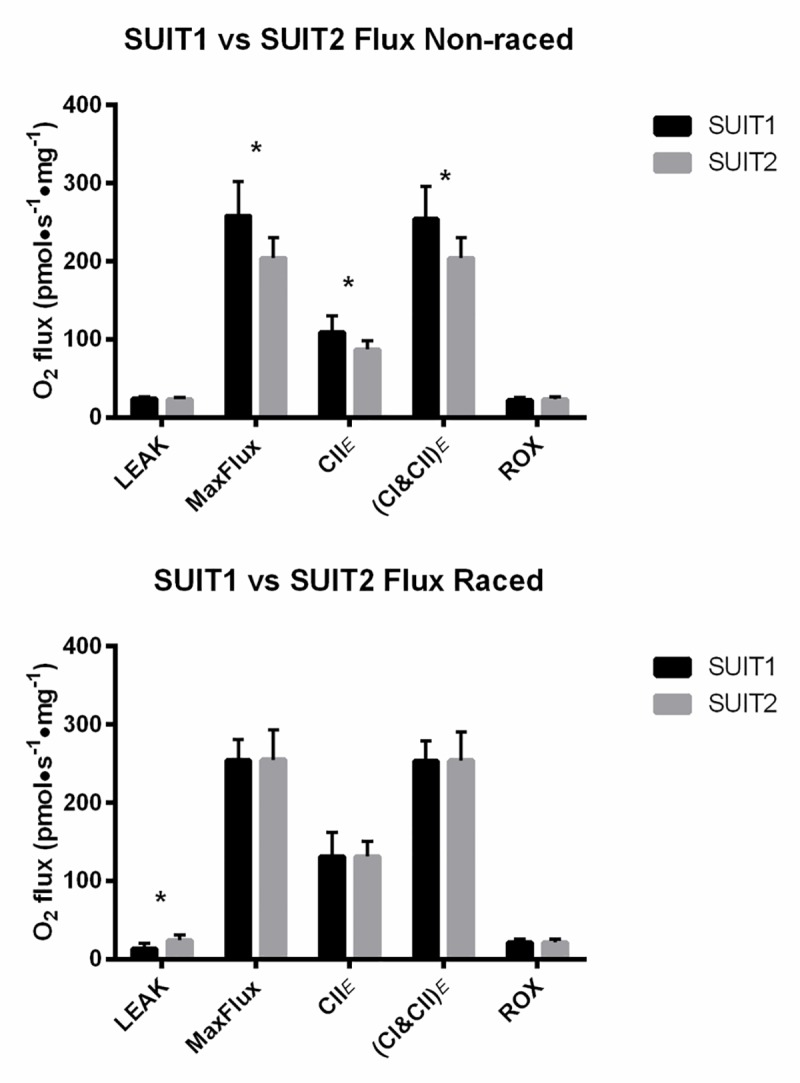
Comparison of SUIT1 versus SUIT2 flux values in the Non-raced (A) and Raced (B) Alaskan Huskies. In panel A, in the Non-raced dogs the combination of fatty acid (FAO) and NADH-linked substrates (SUIT 1) are required to reach the highest flux rates observed in the titration protocol (J_O2max_). However, in the Raced dogs (B), disparities between SUIT1 and SUIT2 are eliminated due to greater oxidation of NADH-derived substrates such that J_O2max_ can be achieved with NADH-linked substrates only. N = 5 (Non-raced) and 6 (Raced). *p<0.05.

The comparison of mitochondrial respiratory fluxes with differing substrate supply (Fig 4) revealed an important difference in substrate utilization between Raced and Non-raced dogs. Raced dogs were able to achieve the same mitochondrial respiratory capacities whether or not FAO was supplied to the electron transfer system. However maximum oxidative phosphorylation capacity (J_O2max_), in Non-raced dogs could only be achieved when electrons were provided by all 3 sources (CI, CII, and electron-transferring flavoprotein).

## Discussion

The major finding in this study is that the extreme exercise included in the 1600 km Iditarod Race resulted in significant upregulation of substrate driven skeletal muscle mitochondrial oxidative phosphorylation and electron transfer system capacity in Alaskan Huskies. The maximum mitochondrial oxygen flux of 254±26 pmol^.^s^-1.^mg^-1^ for OXPHOS capacity (coupled respiration) and 254±37 pmol^.^s^-1.^mg^-1^ for ETS capacity (non-coupled respiration) are among the highest values reported for mammalian species. After racing, these maximal respiratory capacities were achieved whether or not FAO substrates were present. In Raced dogs the mass-specific coupled respiration with mitochondrial NADH-generating substrates for CI increased 36% and maximum oxygen flux (J_O2max_) through the electron transfer system with convergent electron supply with addition of S (SUIT2) was elevated by 25%. Finally, racing increased maximal oxygen flux from NADH-generating substrates, and in support of our previous findings, this seemed to occur concurrently with a decrease in the capacity for utilization of fat derived substrates.

Unique animal model: There is a growing body of literature characterizing Alaskan Huskies because of their impressive endurance exercise capacity and incredible daily energy flux during competition [6–8,17–21]. We have begun to classify these dogs as highly stress resistant because of their adaptability to environmental stress. The stress resistance likely results from strong selective pressure to survive in extreme environmental conditions and for the ability to perform work, which is well documented in lay literature. The Alaskan Husky is a good model to study stress resistance and the mechanism of rapid acclimation because they must be able to simultaneously maintain body temperature in extreme cold, while performing prolonged vigorous exercise.

Maximal flux (J_O2max_): In the current study, maximal oxygen fluxes were determined in permeabilized fibers from the biceps femoris muscle (a muscle that develops marked hypertrophy during the seasonal conditioning of sled dogs and was thus selected to identify conditioning-associated changes on a cellular level). We believe these maximal coupled and non-coupled respiratory fluxes (254±26 and 254±37 pmol^.^s^-1.^mg^-1^, respectively) are the highest mean values recorded in mammalian skeletal muscle. For comparison, human vastus lateralis muscle is known to vary from 60–180 pmol^.^s^-1.^mg^-1^ in sedentary to highly fit individuals [14]. In addition, a study that examined competitive Arabian and mixed-breed racing horses reported average values of 129 and 153 pmol^.^s^-1.^mg^-1^ for coupled and non-coupled respiration, respectively. Therefore, the maximal muscle mass-specific fluxes of Alaskan Huskies far exceed those of competitive horses. We are currently unaware of any studies evaluating mitochondrial respiration in permeabilized fibers of pronghorn antelope perhaps the only other mammal that compares to Alaskan Huskies in aerobic capacity [1].

In a comprehensive review, Poole and Erickson [2] compared the factors that contribute to the high aerobic capacity in both horses and dogs. In this publication a regression line is presented for mass specific mitochondrial volume versus V_O2max_. For most species, including horses, the V_O2max_ can be predicted from mitochondrial volume. Therefore, in most species, an increase in V_O2max_ is commensurate with increases in mitochondrial density following the concept of symmorphosis [22]. However, dogs fall below the regression line indicating that dogs have a higher V_O2max_ than predicted by their mitochondrial volume. The data presented here corroborate those findings in that the dogs have exceptionally high respiration per mg of tissue indicating excess capacity of mitochondria as has also been shown in humans [23]. It is also likely that Alaskan Huskies deviate more from this relationship than other canines, although this has not been directly tested.

Oxidation in the presence or absence of fat substrates (SUIT1 versus SUIT2): In SUIT1 a novel finding was the slight decrease in maximal coupled respiration with only a fatty acid substrate in Raced dogs, which is consistent with decreased whole body fat oxidation during the same absolute submaximal exercise as previously reported [24]. However, this is in contrast to a cross-sectional study of mitochondria from human athletes with different levels of aerobic fitness [25], as well as numerous studies documenting increased fat utilization during exercise in aerobically-trained humans (reviewed in [26]). Addition of substrates to generate NADH for CI increased maximal oxygen flux and did not differ between groups (Fig 2). Importantly, maximal oxygen flux with fat-derived substrates required the addition of NADH-generating substrates. These results are in contrast to those in SUIT2 where maximal NADH-based substrate oxidation was increased in Raced dogs. In addition, overall maximal oxygen fluxes were achievable with only NADH-generating substrates in Raced whereas this was not the case Non-raced. These data indicate that there is an enhancement of NADH-linked (primarily carbohydrate) substrate utilization in Raced and that maximal fluxes can be sustained by carbohydrate alone. Although it has been assumed that Alaskan Huskies rely on fats for energy during prolonged exercise due to the very high amount of dietary fat intake as well as the documented increases in fat utilization during submaximal exercise in other animals, we now have two independent measurements using in vivo whole body oxidation [8] and ex vivo intramuscular mitochondrial respiration that support an increased use of carbohydrates and decreased use of fatty acids after prolonged exercise. Further, our previous studies on giant sarcolemmal vesicles from exercise trained Alaskan Huskies indicate an increase in glucose transport activity [6]. In combination, these data support the notion that the primary adaption of substrate use with exercise training in Alaskan Huskies is the increase the capacity for carbohydrate oxidation, which may be needed to sustain workloads required to run 1600 km at 16 km/h.

Mitochondrial biogenesis and function: In our previous work on mitochondrial adaptations in Alaskan Huskies, we found no evidence of an increase in mitochondrial biogenesis, as determined by mitochondrial protein synthesis, after the initiation of an exercise-training program [5]. Although this result initially took us by surprise given the highly aerobic nature of Alaskan Huskies, comparison with our previous studies in humans indicates that the Huskies maintain resting mitochondrial protein synthesis rates that are 2–3 times greater than humans. In the current study, we further demonstrate that indeed Alaskan Huskies increase substrate specific maximal oxygen flux and phosphorylation capacity increases so that coupled respiration approximates non-coupled flux (P/E approximating the maximum value of 1.0) indicating significant mitochondrial remodeling. Because of these findings, we speculate that the high mitochondrial protein synthesis rates observed in the untrained state allow for rapid remodeling that does not necessitate further increases in protein synthesis after training. It could be argued that it is not energetically efficient to maintain such high turnover rates. However this remodeling pattern allowed for a higher coupled respiration at an unchanged mitochondrial volume, which would enhance mitochondrial performance during exercise without increasing electron leak or heat loss associated with expansion of mitochondrial volume. In this case, selection for adaptability to extreme environmental conditions and energetic demands makes this an acceptable energetic tradeoff.

The relative and absolute values for LEAK respiration were ~10% of maximum respiration and decreased in Raced dogs in SUIT1 but not SUIT2, indicating low endogenous uncoupling and constitutively high mitochondrial efficiency. Uncoupling reduces mitochondrial efficiency and aerobic exercise capacity [27,28] so it is not surprising that this effect is minimal in elite athletes such as the subjects of this study, but like our findings of altered capacity for fatty acid oxidation, decreased LEAK respiration is in contrast to reports of increased LEAK respiration in human athletes with increased aerobic fitness [25]. There are numerous circumstances that could be expected to increase uncoupling in racing sled dogs, including a diet that is approximately 65–70% fat (on a caloric intake basis) [7] as well as living in a very cold environment. Thus, the fact that there was no increase in uncoupling suggests that these stimuli for production of uncoupling proteins were counteracted by the need for high mitochondrial efficiency, consistent with a recently proposed hypothesis for “tissue-specific” coupling that prioritizes ATP synthesis over heat production in working muscle [29].

Access to fully-conditioned elite racing sled dogs like those used in this study is challenging in a prospective research context, and as a result studies–particularly those that necessitate invasive procedures such as muscle biopsy–can suffer from lack of statistical power due to low subject numbers. We reviewed our data to identify specific measurements for which our statistical comparison may have been underpowered (0.05<p<0.2). Our study had a power of 0.8 to detect a 30% difference (7.62 pmol/mg/s) in residual J_O2_ in SUIT 2 between Raced and Non-raced groups. The corresponding measurement in SUIT 1 had a p-value of 0.36, leading to our conclusion that our small sample size did not result in a failure to detect a meaningful effect of conditioning on residual oxygen consumption. Our study had a power of 0.8 to detect a 43% difference (52 pmol/mg/s) J_O2_ supported by electron flow through CII in SUIT 1 between Raced and Non-raced groups. Although this specific comparison arguably was underpowered relative to a meaningful change in oxygen flux, we confirmed statistically significant effect of conditioning for this parameter using SUIT2 so the effect of a small sample size did not affect our conclusions. Finally, our study had a power of 0.8 to detect a 50% difference (67.5 pmol/mg/s) in J_O2_ supported by electron flux through rotenone-sensitive electron flux (i.e., the combination of CI and ETFP). Though there is no specific corresponding endpoint generated in SUIT 1, we do report conditioning-induced changes in both CI-specific J_O2_ (SUIT1) and J_O2_ supported by fatty acid oxidation (SUIT2) as separate endpoints, thus minimizing the effect of low statistical power on the conclusions derived from this study.

Summary: The Alaskan Husky is a unique animal model in which selective breeding has led to the capacity to perform strenuous aerobic exercise for prolonged periods of time. Here we demonstrate that when examined after a 1600 km sled dog race, Alaskan Huskies have peak respiratory capacities that are among the highest mean values recorded in mammalian skeletal muscle. In addition, there are mitochondrial adaptations after extreme exercise that may improve the use of carbohydrate so that maximal fluxes can be achieved in the absence of fatty acid oxidation. Finally, the difference between maximal coupled and non-coupled flux becomes negligible after extreme exercise. From our previous studies [5,8] and the current study, we conclude that Alaskan Huskies maintain high mitochondrial protein turnover to facilitate rapid adaptation to environmental extremes and energetic challenges.

## Supporting information

S1 File Oxygen consumption values for individual subjects and samples reported in this study. (XLSX)
